# Transport of Phage in Melon Plants and Inhibition of Progression of Bacterial Fruit Blotch

**DOI:** 10.3390/v12040477

**Published:** 2020-04-23

**Authors:** Aryan Rahimi-Midani, Tae-Jin Choi

**Affiliations:** Department of Microbiology, Pukyong National University, Busan 48513, Korea; aryanrahimi@pukyong.ac.kr

**Keywords:** phage biocontrol, bacterial fruit blotch, melon, *Acidovorax citrulli*

## Abstract

Bacterial fruit blotch (BFB) is an economically important disease in melons and watermelons for which no effective control method is available. Application of phytobacterium-infecting phage has been evaluated as an alternative means of preventing bacterial diseases in plants. Coating of seeds with bacteriophages infecting *Acidovorax citrulli*, the causal agent of BFB, is effective for controlling the disease, as shown in our previous study. We evaluated the transport of bacteriophage ACPWH from soil to the leaves of melon plants, and we also evaluated its effect on BFB. Leaves of melon plants were spray-inoculated with *A. citrulli*, and bacteriophage ACPWH was added to soil after symptoms had developed. ACPWH was detected by PCR in foliar tissue 8 h after addition to soil. DAPI-stained ACPWH accumulated at the leaf tip after 24 h. Melon treated with ACPWH showed 27% disease severity, compared to 80% for the non-treated control, indicating that ACPWH can be used to control BFB.

## 1. Introduction

Bacterial fruit blotch (BFB), caused by the phytobacterium *Acidovorax citrulli*, is an economically important disease in the cucurbit production industry. *A. citrulli* is a seed-borne bacterium which resides in the seed coat and initiates infection after germination of the plant [1]. BFB can occur at any growth stage in watermelons, melons, and other cucurbits. The symptoms of BFB include water-soaked lesions on cotyledons, hypocotyls, and leaves. Water-soaked lesions on fruit are small and irregular but progress through the rind, resulting in decays and cracks and complete loss of product. Control strategies, including application of antibiotics and chemicals such as peroxyacetic acid and NaOCl, do not eradicate *A. citrulli* [2]. Biological treatments have been proposed for controlling BFB. For instance, soaking of pre-germinated melon seeds in *Bacillus subtilis* suspension showed 61.7% biocontrol efficacy due to surfactin-mediated antibacterial activity [3]. Two rhizobacteria, *Paenibacillus polymyxa* and *Sinomonas atrocyanea,* were able to significantly reduce BFB incidence in watermelon plants when they were added to *A. citrulli* inoculated soil. [4]. BFB represents a threat to the watermelon and melon industry, and, so, more effective control methods are needed.

Despite the ability of bacteriophages (viruses of bacteria) to control bacterial disease, their application was curtailed upon the advent of the antibiotic era [5]. However, the antibiotic resistance crisis caused by overuse of antibiotics has reignited interest in bacteriophages for controlling human [6], foodborne, and plant pathogens such as *Salmonella typhimurium* and *Xylella fastidiosa* [7,8]. Bacteriophages infecting phytobacteria have yielded promising results. For instance, Czajkowski et al. [9] showed that phages are effective against soft rot disease in potato slices by at least 95 %, and Schwarczinger et al. [10] reported that phages significantly reduced multiplication of *Erwinia amylovora* and fire blight symptoms by 84%.

Despite the economic loss caused by BFB, phage biocontrol of this disease is in its infancy. Coating of watermelon seeds with the *Myoviridae-* and *Siphoviridae*-family bacteriophages ACP17 and ACPWH, which infect *A. citrulli*, modulated the appearance and progression of BFB [11,12].

Seed coating is a cost-effective inoculation method for controlling diseases [13]; for example, diseases in soybean and watermelon [11,14]. However, the efficiency of phage application is affected by diverse factors [15]. Phages are susceptible to the effects of pH, UV, and temperature. For instance, Iriarte et al. [16] showed that UV irradiation significantly reduced the efficacy of phages ΦXacm 2004-16 and ΦXV3-16.

Because soil has a better pH and UV range than plants, addition of phages to soil can be used for phage application processes. Phage translocation from soil to plants was reported by Nagy et al. [17], suggesting phage addition to soil has potential for controlling bacterial diseases [18]. However, studies related to translocation are very limited, and more information is necessary for better application of phages.

Melon (*Cucumis melo*) is an important cash crop worldwide. As a member of the family *Cucurbitaceae*, melon is a host of *A. citrulli*, but no commercial cucurbit cultivar with resistance has been introduced [1,19]. Genetically, *A. citrulli* is classified into group I, which mainly infects melon and other non-watermelon cucurbits, and group II, which infects only watermelon [20].

In this study, we evaluated the ability of bacteriophage ACPWH (genetic group I) to control BFB in melons by soil application instead of seed coating. Phage absorption and translocation from soil to the top part of plants were also evaluated.

## 2. Materials and Methods

### 2.1. Bacteria and Phages

*Acidovorax citrulli* strain KACC 17002 (genetic group I) was obtained from the Korean Agricultural Culture Collection and cultured in KB liquid medium overnight at 34 °C. Bacteriophage ACPWH, isolated from a watermelon farm, was used in this study [11]. To propagate ACPWH, the top agar method was employed as described previously [21]. Briefly, 0.3 mL of *A. citrulli* (OD_600_, 0.6, ~ 10^8^ CFU/mL) was mixed with 100 μL of ACPWH (multiplicity of infection, 0.01) and 4 mL of 0.7% KB top agar. The mixture was overlaid on 1.5% KB agar and incubated overnight. Next, 10 mL of SM buffer was added and gently shaken to distribute phages evenly. The mixtures were pooled, crushed, and centrifuged at 2700× *g* for 30 min to remove debris. The supernatant was passed through a 0.2 μm filter, the titer was determined by plaque assay, and the samples were stored at 4 °C until required.

### 2.2. Fluorescent Staining and Detection of Phage

Bacteriophage ACPWH, prepared as above (10^9^ PFU/mL), was mixed with 4′,6-diamidino-2-phenylindole (DAPI, Sigma Aldrich, St. Louis, MO, USA) to a final concentration of 1 μg/mL. Bacteriophage solution was incubated in the dark for 10 min and dialyzed to remove unabsorbed stain, as described previously [22].

Commercial melon seeds (Nongwoo Bio Co, Suwon, Korea) were purchased from a market and surface sterilized with 10% sodium hypochlorite for 20 min. One seed was sown per plastic nursery pot (top, 10 cm diameter; bottom, 8 cm diameter; height, 12 cm) containing 200 g of sterilized autoclaved soil. Pots were kept in a room under 12/12 h light/dark conditions at 35 °C with 90% relative humidity for 2 weeks until the seedlings had four true leaves. To enhance absorption, scratches were made using a single-edge blade on the primary root right before the secondary roots. Then, 150 mL of stained phage (10^9^ PFU/mL) was added to each pot. Sterile distilled water was added to the other pots as the negative control. After 24 h, the plants were observed under an HL34T hand lamp (Clare Chemical Research, Montezuma, CO, USA) and photographed with an SLR camera equipped with a fluorescence filter.

### 2.3. Titration of Phage in Plant Tissues

Melon plants were grown and prepared for phage addition, as described above. Phage was added to soil at 7.0 × 10^8^ PFU/g soil. Leaves were collected from the same location every 8 h from 11 sets with three replications. Two grams of leaf tissue was ground with 2 mL of distilled water, using a mortar and pestle. Ground tissue was centrifuged for 30 min at 3000× *g* to remove plant debris, and the supernatant was subjected to titration, as described previously [23].

### 2.4. Detection of ACPWH in Plant Tissues

Melon plants were grown as described above, and phage was added to the soil at 7.0 × 10^8^ PFU/g soil. Five sets with three replications were used, and leaf tissues were collected from the same location at 2, 4, 8, 12, and 24 h after phage addition. In addition, 5 g of tissue from leaf, petiole, and stem was collected 8 h after phage addition. Collected tissues were surface sterilized with 10% sodium hypochlorite for 20 min and rinsed three times with sterile distilled water. DNA was extracted using a Plant DNA Extraction Kit (2NCBIO, Daejeon). Extracted DNA was used as the template for PCR amplification of an 867 bp DNA fragment using an ACPWH capsid protein gene specific forward primer (5′-ATGATCGATGCCTTGGGGTC-3′) and reverse primer (5′-CTCGGCGACAAAGGTCTCTT-3′). The PCR conditions were 35 cycles of denaturation at 95 °C for 30 s, annealing at 55 °C for 20 s, and extension at 72 °C for 20 s, using rTaq Plus 5× PCR Premix (Elpisbio, Daejeon, Korea).

### 2.5. Artificial Inoculation and Phage Treatment

Melon plants were grown for 2 weeks until they had four true leaves. The pots and soil of 100 pots were covered with aluminum foil to avoid cross contamination and spray-inoculated with 20 mL of *A. citrulli* solution (10^7^ CFU/mL) on the top side of the leaves. The plants were incubated for 4 days at 35 °C until the appearance of disease symptoms; at that time, phage ACPWH was added to the soil to a final concentration of 10^8^/g soil. Negative-control plants were given sterile distilled water; the experiments were repeated three times. Disease symptoms were scored and data were recorded, according to the standard classification evaluation system for BFB described by Bahar et al., [24] (0–5 scale): 0, no incidence; 1, 1–25% leaf area with yellow lesions; 2, 26–50% leaf area with lesions; 3, 51–75% leaf area with dark brown lesions; and 4, 76–100% leaf area with dark lesions and necrosis. Disease severity was recorded after 10 days, and the survival rate was recorded after 20 days. Disease severity and survival rate were calculated using the formula below [25,26]. Data were analyzed by Student’s *t*-test using GraphPad Prism software (ver. 8) [27].

(1) $$\mathrm{Disease}\;\mathrm{severity}\;\mathrm{index}\;=\frac{\mathrm{Sum}\;\mathrm{of}\;\mathrm{all}\;\mathrm{disease}\;\mathrm{rating}}{\mathrm{Total}\;\mathrm{number}\;\mathrm{of}\;\mathrm{rating}\;\times \;\mathrm{maximum}\;\mathrm{disease}\;\mathrm{grade}}\;\times \;100$$

(2) $$\mathrm{Survival}\;\mathrm{rate}\;=\frac{\mathrm{Number}\;\mathrm{of}\;\mathrm{survived}\;\mathrm{plants}}{\mathrm{Total}\;\mathrm{number}\;\mathrm{of}\;\mathrm{plants}\;\mathrm{on}\;\mathrm{the}\;\mathrm{first}\;\mathrm{day}\;}$$

## 3. Results

### 3.1. Detection of Phage Translocation by PCR

**Figure 1:**
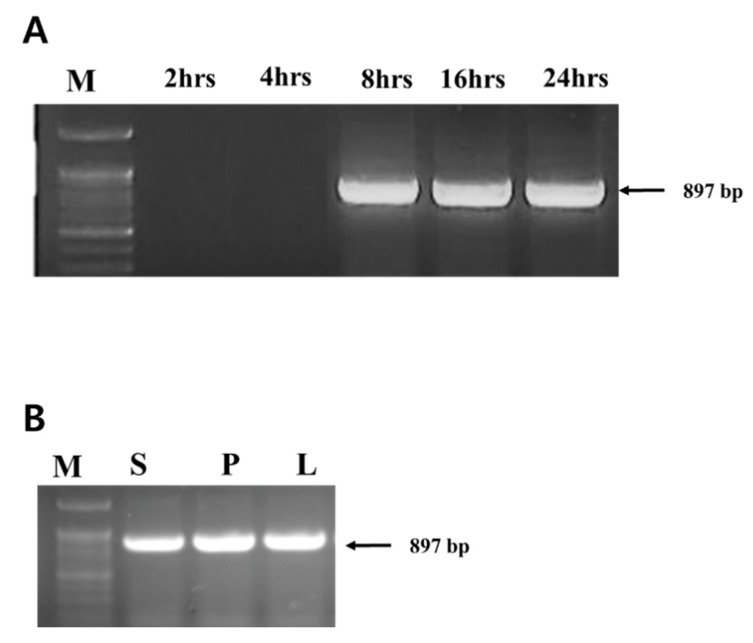
Detection of phage ACPWH in melon tissues. DNA was extracted from leaf tissues collected 2 h, 4 h, 8 h, 16 h, and 24 h post-inoculation of phage to soil (**A**), tissues 8 h post-inoculation (**B**), and amplified by PCR. M, 100 bp marker; S, stem; P, leaf petiole; L, leaf blade.

PCR was performed on plants treated with bacteriophage ACPWH but no additional bacterial treatments. Translocation of phage ACPWH was traced by PCR of extracts of top leaves up to 24 h after phage addition. ACPWH was not detected in leaf tissue 2 h and 4 h after addition, but was detected from 8 h to 24 h post-inoculation (Figure 1A). The phage genome was also detected in the stem and petiole 8 h after addition to soil (Figure 1B).

### 3.2. Phage Titer on Leaves

**Figure 2:**
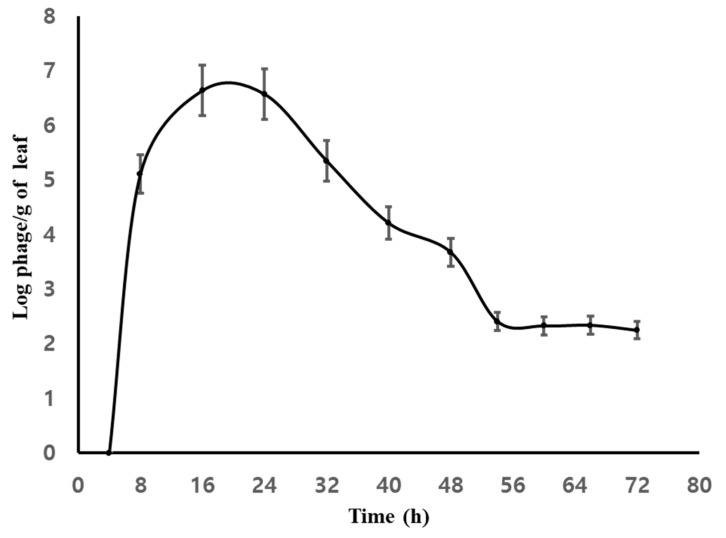
Quantitation of phage ACPWH translocated from soil to leaves. Melon leaves were collected every 8 h after addition of phage to soil and subjected to plaque assay. The results are means of three replications; vertical lines are standard deviations.

Phage ACPWH was added to the soil of melon plants with root damage to a final concentration of 7 × 10^8^ PFU/g soil without *A. citrulli* treatment. At 8 h post-inoculation, the phage titer was 6.3 × 10^8^ PFU/g soil. After 8 h, ACPWH was detected in the top four leaves at 1.5 × 10^5^ PFU/g melon leaf tissue (Figure 2). The phage titer peaked at 6.5 × 10^6^ PFU/g melon leaf after 16 h and, subsequently, declined to 2.9 × 10^3^ PFU/g melon leaf after 56 h, which was maintained until 72 h after addition (Figure 2).

### 3.3. Tracing of Fluorescent Phage on Leaves

**Figure 3:**
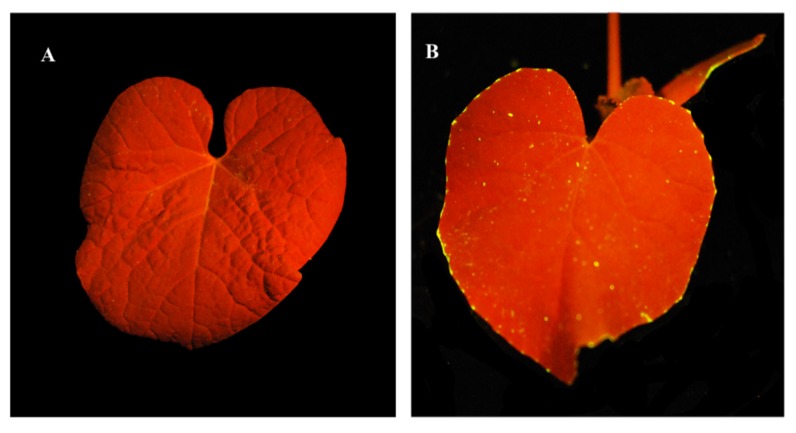
Fluorescence images of translocation to the leaves of bacteriophages added to soil. (**A**) Melon plant without phage addition. (**B**) Melon plant 24 h after addition of DAPI-stained phage ACPWH to soil.

Translocation of DAPI-stained phage was traced, following its addition to soil without *A. citrulli* inoculation. Stained ACPWH was added to soil at 7 × 10^8^ PFU/mL and imaged under a UV lamp after 24 h. Compared to the phage-free control, fluorescent phage accumulated on treated leaves (Figure 3). Fluorescent specks were observed throughout the leaf blade, but fluorescent phage accumulated principally at the leaf margin (Figure 3B).

### 3.4. Control Effect of Translocated Phage

**Figure 4:**
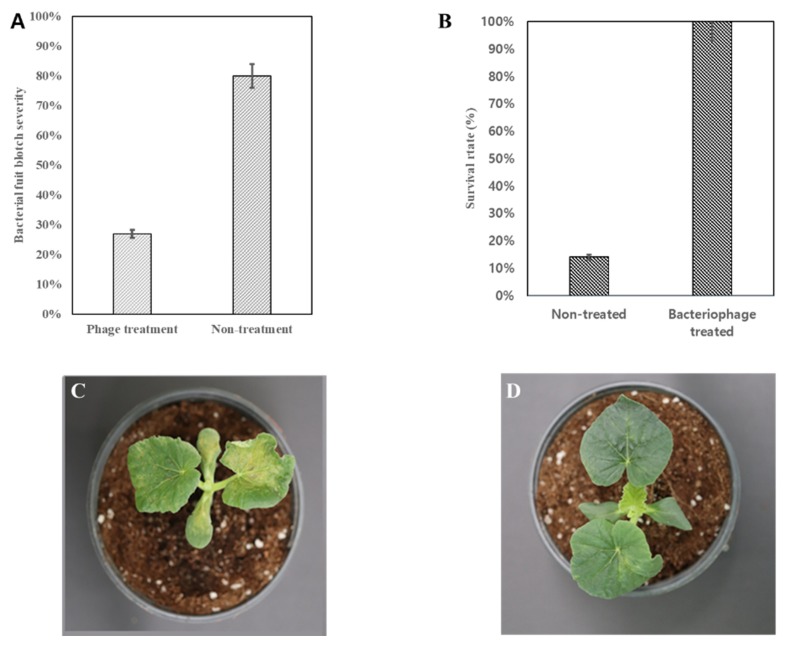
Effect of bacteriophage ACPWH against BFB on melon plants. BFB was induced by artificial inoculation of *A. citrulli*; ACPWH was added to soil 3 days later. (**A**) Disease severity 10 days after phage treatment. (**B**) Survival rate 20 days after treatment. (**C**) Melon plant inoculated with *A. citrulli* and not treated. (**D**) Melon plant inoculated with *A. citrulli* and treated. Results in (A) and (B) are the means of three replications; vertical lines are standard deviations.

Melon plants sprayed with *A. citrulli* started to show disease symptoms, 4 days post-inoculation; at this time, phage ACPWH was added to the soil. Disease progression was monitored, and the incidence and severity were measured 20 days after phage application. Because phage was added to the soil of plants with BFB symptoms, the disease incidence was 100% in the treated and non-treated groups. However, the disease severity in the phage-treated group was 27%, compared to 80% in the non-treated group (Figure 4A). The survival rate was 100% in the phage-treated group, compared to 14% in the non-treated group (Figure 4B). Furthermore, the surviving plants in the non-treated group showed typical signs of BFB, such as light brown and water-soaked lesions on leaves (Figure 4C). By contrast, BFB symptoms cleared in the treated group, 20 days post-treatment (Figure 4D).

## 4. Discussion

With the difficulties in controlling bacterial diseases using conventional strategies and the appearance of antibiotic resistant bacteria in some economically important plant diseases, biocontrol with bacteriophages is receiving more attention these days. Bacteriophages have several potential advantages for use in disease control. They are natural components of the biosphere and can be isolated wherever bacteria are present, but they are also self-limiting because they are quickly degraded in the absence of their hosts. They are highly discriminatory and infect only the target bacteria; they are not harmful to eukaryotic cells and other members of the indigenous flora. Additionally, their preparation, storage, and application is fairly easy [28]. Available methods to control *A. citrulli* are not sufficient, and overuse of pesticides has raised public concern, increasing the importance of biological control methods. Seed treatment has been proposed as a control strategy for *A. citrulli*. For instance, heating and HCl treatment of watermelon seeds protected against *A. citrulli* but also reduced the germination rate and affected plant health [29,30]. Treatment of watermelon or melon seeds with a biocontrol agent such as non-pathogenic *A. citrulli* [31], *Bacillus subtilis* [3], *Paenibacillus polymyxa* and *Sinomonas atrocyanea* [4], or *Rhodotorula glutinis* [32] is effective against *A. citrulli*.

Recently, we have isolated two *A. citrulli* infecting bacteriophages named as ACP17 and ACPWH, both of which enhanced plant germination and survival when watermelon seeds were coated with these phages [11,12]. In particular, phage ACPWH, used in this study, showed a wider host range and infected 39 of 42 *A. citrulli* strains tested, including 10 strains that were resistant to phage ACP17 but did not infect other non-host bacteria. The host specificity to *A. citrulli* and a wide host range within the species make ACPWH a good candidate as a seed coating agent alone or with other phages.

Although phage ACPWH was proven to be effective for seed coating and, thereby, prevention of BFB at the early stage, BFB can develop at a later stage by contamination from contaminated soil, workers, and tools and can infect the foliar part [1,2]. Phages can be sprayed on leaves after BFB symptom development. However, phages are sensitive to UV, so reducing their protective effect and designing strategies for reducing exposure of bacteriophages to UV is critical to the use of phages as biological control agents [16]. Therefore, application of phages in water or protective formulations into soil has been evaluated [33].

Following addition to soil, translocation of phage to the upper part of melon plants was assessed by PCR and plaque assay. Bacteriophage was detected in various parts of plants 8 h after application, and its abundance increased thereafter (Figure 1). Similarly, more phage was detected in leaf tissue at 16 h, compared to 8 h after addition (Figure 2). The time for translocation to the leaf tissue and duration on the leaf tissue can be affected by the phage, plant species, plant size, and soil condition. For example, Iriarte et al. [24] reported that phages ΦMI2 and ΦRS5 added to the soil of the tomato were detected after 4 h and 24 h, respectively, and remained detectable for 1 week [34].

After peaking 16 h and 24 h after addition to the soil, the phage titer decreased until 56 h and was maintained thereafter (Figure 2). A decrease in phage titer in plant tissue has also been reported by others. For example, the titer of bacteriophage F2 added to the damaged root of bean and corn plants dropped 7 days post-inoculation [35]. Additionally, the titer of a phage mixture in a tomato plant dropped from 10^5^ PFU/g plant tissue to undetectable within 1 week after inoculation because of the lack of bacteria in the damaged root [34]. The phage titer in soil remained unchanged up to 72 h after addition (Figure S1A). Additionally, additional uptake of phage from damaged root tissue I was inhibited by the healing process. As shown in Supplementary Figure 1B, phage ACPWH was not inactivated by melon extract. Indeed, phages are reportedly stable in extracts of Chinese keys (*Boesenbergia rotunda*), oroxylum (*Oroxylum indicum*), and *Stephania suberosa* for 24 h [36], possibly as a result of removal by guttation. Indeed, after 24 h, most phages were at the edge of the melon leaf, where guttation occurs (Figure 3).

We introduced an artificial scratch on the root to enhance phage absorption by plants. Enhancement of phage absorption has been reported in corn, bean, and tomato [35,37]. By contrast, addition of phages to soil, without damaging the root, was effective against pathogen bacteria in, for example, calla, tomato, and carrot [38,39,40,41]. At the start, we show partial protection by addition of phage to soil, without damaging the root, but damaging of the root part improved the translocation. In field conditions, root damage can be done with tools such as gardening trowels before addition of phage to soil, which also helps close contact between the phage and root.

Translocation and accumulation of phage in leaf tissue was visualized by DAPI staining (Figure 3). Although phages were detected in leaf blade, their accumulation was greater at the marginal region of the leaf. This indicates that the phages translocated through the xylem with water from soil.

The therapeutic activity of phages was assessed by adding phage to plants with BFB symptoms after artificial inoculation. Melon plants treated with phage ACPWH, by adding this phage to soil, showed only 20% disease severity, compared to 80% in the control (Figure 4A). In addition, the BFB symptoms did not progress. This indicates that the phage translocated from soil to leaf tissue and killed infecting bacteria, halting symptom progress. Although coating of seed with bacteriophage was effective for the prevention of BFB [11], BFB can occur at a later stage of plant growth, which can be treated by adding the phage to soil, as proven in this study.

## Supplementary Materials

The following are available online at https://www.mdpi.com/1999-4915/12/4/477/s1, Figure S1: Stability of phage ACPWH in soil and melon extract. Phage ACPWH was added to soil at an initial titer of 3.3 × 10^7^ PFU/g soil.
