# Supplementary material for: Transport of Phage in Melon Plants and Inhibition of Progression of Bacterial Fruit Blotch
Source: Viruses. 2020 Apr 23;12(4):477. doi: 10.3390/v12040477 (PMC7232510; doi:10.3390/v12040477)
Supplement: Supplementary file 1 [file viruses-12-00477-s001.pdf]

Supplementary Data

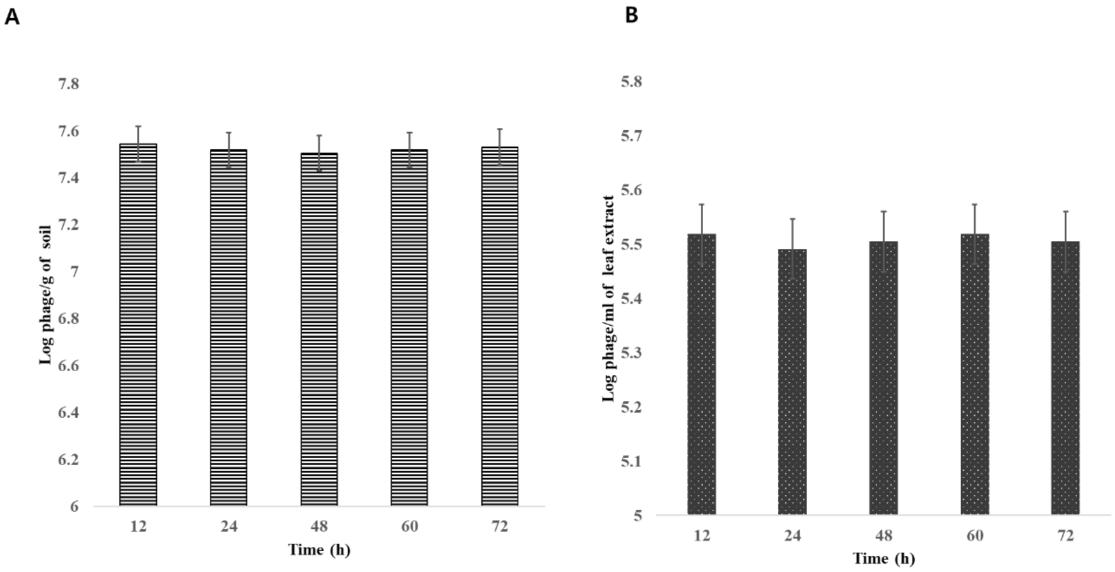

Figure 1. Stability of phage ACPHW in soil and melon extract. Phage ACPWH was added to soil at an initial titer of 3.3×10^7 PFU/g soil (**A**) or 3.3×10^5 PFU/mL melon extract (**B**) and titers were determined by plaque assay. The results are means of three replications; vertical lines are standard deviations.
